# An ensemble model approach for identifying pulmonary tuberculosis on chest X-ray

**DOI:** 10.3389/fmed.2026.1903513

**Published:** 2026-08-28

**Authors:** Weiying Qin, Qingxin Hu, Jing Jing

**Affiliations:** 1 Department of Radiology, Affiliated Hospital of Integrated Traditional Chinese and Western Medicine, Nanjing University of Chinese Medicine, Nanjing, China; 2 Jiangsu Province Academy of Traditional Chinese Medicine, Nanjing, China

**Keywords:** chest x-ray, classification, deep learning, model ensemble, pulmonary tuberculosis

## Abstract

Tuberculosis (TB) is a global infectious disease, and chest x-ray (CXR) examination is a primary screening method. However, manual image interpretation can suffer from low diagnostic efficiency, poor consistency, and limitations imposed by the experience of the interpreters, necessitating the development of intelligent identification tools. This study aims to construct and validate a deep learning ensemble model-based CXR image recognition system for TB, achieving accurate and intelligent screening. The Shenzhen TB CXR public dataset was used as the training dataset, randomly divided into training and test datasets; the Montgomery public dataset was used as an independent external validation dataset. Nine convolutional neural network models (DenseNet201, EfficientNetB7, EfficientNetV2S, InceptionResNetV2, MobileNetV3Large, NASNetLarge, ResNet50V2, VGG19, and Xception) were built using the PyTorch framework and benchmarked. The optimal model was selected using the F1 score as the core metric. Furthermore, using the raw scores of the individual models as input features, four ensemble strategies—linear regression, logistic regression, performance-weighted averaging, and eXtreme Gradient Boosting (XGBoost)—were constructed to evaluate the classification performance and generalization ability of each model. The results show that all nine individual models can distinguish between normal and TB CXR images (all *P* < 0.001). The VGG19 model performed best on the independent validation dataset, with an area under the curve (AUC) of 0.853 and an F1 score of 0.793. The ensemble models further improved classification performance. The performance-weighted averaging ensemble model achieved an AUC of 0.880 and F1 score of 0.861 on the independent validation dataset, outperforming the optimal individual model and other ensemble strategies. The performance-weighted averaging ensemble model constructed in this study can accurately identify pulmonary TB CXR images and has high generalization performance. It can provide an intelligent tool for primary-level pulmonary TB screening. In the future, it can be further prospectively validated in multi-center, large-sample clinical datasets to promote its clinical translation and application.

## 1 Introduction

Tuberculosis (TB) is a chronic respiratory infectious disease caused by Mycobacterium tuberculosis. It is one of the leading causes of death from a single infectious disease globally, posing a challenge to global public health security (1). The World Health Organization's Global Tuberculosis Report shows that in recent years, the number of new TB cases worldwide has consistently remained above 10 million, with over one million deaths attributed to TB. Low- and middle-income countries account for over 90% of the disease burden (2). Chest x-ray (CXR) examination is convenient, inexpensive, and widely available, making it the preferred imaging method for primary screening and initial screening of suspected TB cases globally (3).

In clinical practice, the CXR imaging manifestations of TB are complex and diverse, exhibiting various signs such as exudation, nodules, cavitation, and fibrosis. Diagnostic accuracy highly depends on the clinical experience and professional expertise of the radiologist. However, in grassroots areas with relatively scarce medical resources, there is a significant shortage of radiologists with extensive experience in interpreting images. Manual image interpretation can lead to problems such as low diagnostic efficiency and poor consistency among interpreters, especially for early and atypical cases of pulmonary TB (PTB), where the limitations of manual diagnosis are more pronounced (4). Furthermore, large-scale PTB screening programs generate a heavy workload for radiologists due to the massive volume of CXR images, further exacerbating fluctuations in diagnostic quality (5, 6). Therefore, developing accurate and widely applicable intelligent CXR image recognition tools for PTB is of significant clinical and public health value in addressing the shortage of grassroots medical resources and improving the efficiency and diagnostic consistency of PTB screening.

In recent years, artificial intelligence (AI) technology, with deep learning at its core, has achieved breakthroughs in the field of medical image analysis, demonstrating excellent performance in CXR image recognition for various chest diseases such as lung tumors, pneumonia, and TB (7). Convolutional Neural Networks (CNNs), as the core model of deep learning in computer vision, can automatically extract deep features from images, overcoming the limitations of traditional machine learning methods that rely on manual feature engineering, and possess unique advantages in medical image classification tasks. Existing research has confirmed that various CNNs models, such as DenseNet, EfficientNet, and ResNet (8), can effectively classify and recognize CXR images of TB (9), and the diagnostic performance of some models has reached the level of senior radiologists, demonstrating potential for clinical application (10, 11).

Despite the achievements of existing research, many problems and research gaps remain. First, most current studies rely solely on single-center datasets for model training and validation, lacking independent external datasets for generalization performance evaluation. This may limit the effectiveness of models in real-world clinical scenarios (12). Medical imaging data varies due to differences in equipment, population groups, and acquisition procedures. Models that perform well on single-center datasets often experience significant performance degradation on external data, hindering clinical translation (13). Second, existing research largely focuses on optimizing and applying single deep learning models. However, single models have inherent performance bottlenecks. Different CNN models emphasize different aspects of feature extraction, making it difficult for a single model to capture the complex signs in PTB CXR images, resulting in insufficient robustness and generalization (14). Furthermore, existing research has not fully explored the application of model ensemble strategies in PTB CXR identification, particularly the value of ensemble model strategies in this scenario. The ensemble model can effectively integrate the predictive advantages of multiple base models while avoiding the overfitting risk of a single model. It can improve model classification performance and is expected to further enhance the model's generalization ability. However, research on federated learning ensemble models for CXR identification of PTB is still relatively scarce (15). Furthermore, existing research lacks benchmark performance comparisons of various mainstream CNNs models and head-to-head comparative analyses of the advantages and disadvantages of different ensemble strategies, failing to provide reliable methodological references for model selection in this field (16–18).

To address the aforementioned research gaps, this study constructed a deep learning-based recognition and validation system for pulmonary TB CXR images. First, benchmark tests were conducted on nine CNN models (DenseNet201, EfficientNetB7, EfficientNetV2S, InceptionResNetV2, MobileNetV3Large, NASNetLarge, ResNet50V2, VGG19, and Xception) to evaluate their classification performance on the training dataset, internal test dataset, and independent external validation dataset, clarifying the strengths and weaknesses of different models in the TB CXR recognition task. Based on this, using the raw output scores of the nine individual models as input features, four ensemble models linear regression, logistic regression, performance-weighted averaging, and eXtreme Gradient Boosting (XGBoost)—were constructed. The performance improvement effects of different ensemble strategies were compared, and the optimal TB CXR recognition model was selected.

The innovations of this study are mainly reflected in three aspects. First, the generalization performance of the model was verified using an independent external dataset; Second, the benchmark performance of nine mainstream CNN models was compared, providing a reference for model selection in the CXR identification task of PTB; Third, the performance of four different ensemble strategies was evaluated, providing new ideas for model optimization in this field. The results of this study can provide an efficient, accurate, and scalable tool for primary-level PTB screening, and also provide a methodological reference for the construction and optimization of deep learning models for medical image classification tasks.

## 2 Materials and methods

### 2.1 Data source and preprocessing

This study incorporated two validated publicly available CXR datasets of PTB, serving as the source dataset for model training and the independent external validation dataset, respectively. The Shenzhen PTB CXR dataset (19), containing 662 CXR images (336 positive cases of PTB and 326 normal controls), was used as the core dataset for model training. It was stratified and randomly split in an 8:2 ratio. The training subset was used for weight training and parameter updates, while the internal test subset was used for performance monitoring and preliminary validation during model training. Both subsets maintained the same percentage of positive samples as the original dataset. The Montgomery PTB CXR dataset served as the independent external validation dataset (20), containing 138 CXR images (58 positive cases of PTB and 80 normal controls). This dataset was not used for model training, parameter tuning, or feature selection; it was only used to evaluate the final model's generalization performance.

This study builds a custom Dataset class based on the PyTorch 2.11.0 framework to complete the entire process of image data preprocessing, covering core steps such as image loading, format conversion, data augmentation, and tensor output. The standardized image preprocessing workflow is as follows: First, images are read using OpenCV-python 4.9.0.80 and Pillow 10.2.0. All single-channel grayscale images are uniformly converted to three-channel RGB format to ensure the consistency of input data format. Then, the images are standardized, scaled, and centered to be uniformly adjusted to an input size of 224 × 224 pixels to adapt to the input requirements of convolutional neural networks. Finally, the image data is converted into floating-point tensors using the ToTensor method, and pixel values are normalized to output tensor data with dimensions [3,224,224], matching the input format of the PyTorch framework. For the training subset, an additional random horizontal flipping data augmentation operation is applied to increase the diversity of the training data and reduce the risk of model overfitting; the internal test subset and the independent external validation dataset do not perform any data augmentation operations, but only complete the standardized preprocessing process.

The data loading process utilizes the tf.data. Dataset module of TensorFlow 2.15.0 to build an efficient data loader. The batch size is uniformly set to 16, and data prefetching and multi-threaded loading mode are enabled to improve data reading efficiency and model training speed. Data shuffling is enabled on the training subset to break the original order of samples and prevent the model from learning irrelevant sequential features. Data shuffling is disabled on the internal test dataset and the independent external validation dataset.

### 2.2 Benchmarking of a single deep learning model

This study selected nine CNNs models widely used in medical image analysis and conducted benchmark performance tests. All models were trained using pre-trained weights from the ImageNet dataset and employed transfer learning. The nine CNNs models included are: DenseNet201 (Densely Connected Convolutional Networks 201), EfficientNetB7 (Efficient Convolutional Neural Network B7), EfficientNetV2S (Efficient Convolutional Neural Network V2 small), InceptionResNetV2 (Inception-Residual Convolutional Neural Network V2), MobileNetV3Large (Mobile Convolutional Neural Network V3 Large), NASNetLarge (Neural Architecture Search Network Large), ResNet50V2 (Residual Neural Network 50 V2), VGG19 (Visual Geometry Group 19-layer Convolutional Neural Network), and Xception (Extreme Inception Convolutional Neural Network). All models used a consistent training strategy, hyperparameter settings, and evaluation process.

The training process for each individual model was as follows: (1) Model initialization: The model structure is built based on the PyTorch framework, the pre-trained weights are loaded to initialize the feature extraction layer, and the model output layer is modified to a single neuron fully connected structure to adapt to the output requirements of the TB binary classification recognition task; (2) Hyperparameter settings: The Adam optimizer is selected as the optimizer, the weight decay coefficient is set to 10 × 10^−4^, and the initial learning rate is set to 10 × 10^−4^; the learning rate scheduling strategy adopts the cosine annealing algorithm, T_max is set to 50, and the minimum learning rate is reduced to 1 × 10^−6^; the loss function is selected as the binary cross-entropy loss (BCELoss), adapted to the optimization needs of binary classification tasks; the total number of training epochs is fixed at 30 epochs; (3) Model training: each training epoch strictly follows the process of gradient clearing → forward propagation → loss calculation → back propagation → parameter update; (4) Model inference and label determination: the original tensor output by the model is reduced in dimension and then converted into a predicted probability value in the 0–1 interval through the Sigmoid activation function; with 0.5 as the classification threshold, a predicted probability > 0.5 is judged as positive for PTB, and ≤0.5 is judged as normal; (5) Model evaluation and weight saving: after each training epoch, model inference is performed on the training subset and the internal test subset respectively, and the accuracy, precision, recall, F1 score and area under the receiver operating characteristic (ROC) curve are calculated and statistically analyzed. (6) Final performance evaluation: based on the saved optimal weights, batch inference is completed on the training subset, internal test subset and independent external validation dataset respectively, and the raw prediction score, prediction probability and classification label of each sample are output to complete the performance evaluation of the whole process.

Nine base models employs an online data augmentation strategy using deep learning for medical images. During training, each batch of images undergoes real-time random rotation (±15°), horizontal flipping, random center cropping (cropping ratio 0.8–1.0), and random brightness/contrast fine-tuning (±10%). In each training epoch, the original images generate one augmented variant, with no duplicate augmented samples. The equivalent augmented training sample size in this study was 662 × 30 = 19,860 images. Augmented samples are not included when calculating model performance metrics. All single-model training and inference were performed using graphics processing unit (GPU) acceleration. The compute unified device architecture (CUDA) Toolkit version was 12.6, and the CUDA Deep Neural Network library (cuDNN) version was 8.9.7. NVIDIA GPU CUDA acceleration was enabled, significantly improving model training and inference efficiency. Model building and training were also compatible with the Keras 3.0 framework, ensuring the reproducibility of the model structure. Data processing and metric calculations were performed using NumPy 1.26.3, Pandas 2.2.0, and scikit-learn 1.4.0.

### 2.3 Construction and evaluation of ensemble models

To further improve model performance and enhance the classification performance and generalization ability of the PTB CXR identification model, this study constructed four different ensemble learning models based on the results of single-model benchmark tests, and evaluated the performance improvement effects of different ensemble strategies. The ensemble strategies uses the raw prediction scores output by nine basic deep learning models as input features to construct four ensemble models: Linear Regression, Logistic Regression, performance-weighted averaging, and XGBoost, respectively, to complete the final classification and identification of PTB CXR images.

The construction strategies of the four different model ensemble strategies are as follows: (1) Linear regression model: linear fitting model, which uses the original score of the single model as the independent variable and the true label of the sample as the dependent variable to complete the linear fitting and output the final prediction probability; Linear regression learns the optimal linear ensemble of predictions from each base model by minimizing the mean squared error, which is equivalent to an interpretable linear classification decision. This method is computationally efficient and can combine a number of input features to give a transparent fusion rule. Therefore, it can be incorporated into ensemble strategies as a baseline to measure the gain brought by model ensemble. (2) Logistic regression model: binary classification linear model, which completes the nonlinear transformation of features through the Logistic function, adapts to the output requirements of binary classification tasks, and is the widely used ensemble model in medical image classification tasks; (3) Performance-weighted averaging model: adopts the improved weighted averaging algorithm, which uses the AUC value of each single model on the independent validation dataset as the weight to perform weighted averaging on the prediction probability of the single model. The weight is positively correlated with the generalization performance of the single model. When the total AUC is 0, it degenerates into equal weighted mean aggregation; (4) XGBoost model: extreme gradient boosting tree model, which builds an ensemble tree model based on the gradient boosting framework, which can capture the nonlinear relationship between features of different single models and achieve the optimization of prediction performance.

This study constructed four ensemble models, using the original predicted scores output by nine basic deep learning models as input features. The linear regression ensemble directly uses LinearRegression from Scikit-learn 1.4.0 to fit the training set features and binary labels without adding a regularization term, generating continuous predicted values by minimizing the mean squared error. The logistic regression ensemble uses Logistic Regression, setting L2 regularization and a penalty coefficient C = 1.0, with a fixed maximum number of iterations of 1,000. The model directly outputs the probability estimate of a sample belonging to the tuberculosis-positive category. The performance-weighted averaging strategy uses the AUC obtained by each base model on the internal test dataset as weight coefficients, linearly weighting and summing the nine original predicted scores, then converting them into the final predicted probability using the Sigmoid function. After weight normalization, the sum is ensured to be 1. The XGBoost ensemble model implements a classifier mode using the XGBoost library. Key parameters are set as follows: maximum tree depth 3, learning rate 0.1, number of weak learners 100, subsampling ratio 0.8, and feature sampling ratio 0.8. The objective function is defined as binary logistic regression. Logistic loss is used as the evaluation metric, and the label encoder is disabled to avoid redundant transformations. The output prediction values of the four ensemble models are divided into final categories with a threshold of 0.5 (>0.5: TB; ≤0.5: normal). The feature matrices of the four combined models are constructed from the prediction scores generated from the training set. All models undergo final performance evaluation on independent external validation dataset.

The training and evaluation process for ensemble models is consistent with that for single-model benchmarking: the raw scores of the single models in the training subset are used as training data to complete the training and parameter fitting of the ensemble model; the internal test subset data is used to perform preliminary validation of the model's performance; and the independent external validation dataset data is used to perform the final evaluation of the model's generalization performance. The classification threshold is uniformly set to 0.5; a prediction probability > 0.5 is considered positive for PTB, and ≤0.5 is considered normal (negative). The core evaluation metric is consistent with that for single models, using the F1 score on the independent external validation dataset as the core metric to compare the performance differences between different ensemble models, and simultaneously compare the performance of the ensemble model with the best single model to verify the effectiveness of the ensemble strategy.

After completing the ensemble model, feature importance analysis was performed to quantify the relative contribution of input features to the ensemble model. This method treats the predictions of different individual models as a set of input features and calculates normalized scores to quantitatively indicate which individual model feature is most valuable for the current task. Specifically, after fitting the ensemble model, the coefficients or importance of the ensemble model are called to obtain the basic contribution, and then the obtained scores are normalized and finally visualized.

The construction and evaluation of linear regression, logistic regression, and performance-weighted averaging models were completed using scikit-learn 1.4.0, and the XGBoost model was implemented using XGBoost 1.7.5.

### 2.4 Statistical analysis and results visualization

Statistical analysis in this study was performed using SciPy 1.12.0. Normality was tested using the Shapiro–Wilk test, and differences between two independent samples were compared using the Mann–Whitney *U* test. A *p*-value < 0.05 was considered statistically significant, and a *p*-value < 0.001 was considered extremely statistically significant. The 95% confidence interval (CI) of the AUC was calculated using the bootstrap method, and the number of repeated samples was set to 1,000.

Visualization of all results was performed using Matplotlib 3.10.0 and Seaborn 0.13.2, including box plots, bar charts, and ROC curves. This study was developed entirely using Python 3.12.1, and all experiments were conducted on a workstation equipped with an NVIDIA GeForce RTX 3090 GPU (24GB Video Random Access Memory), an Intel Core i9-13900 CPU, and 32GB of RAM, running Windows 10 22H2. The flowchart of the methods used in this study is shown in Supplementary Figure 1.

## 3 Results

### 3.1 Performance of single deep learning models

**Figure 1:**
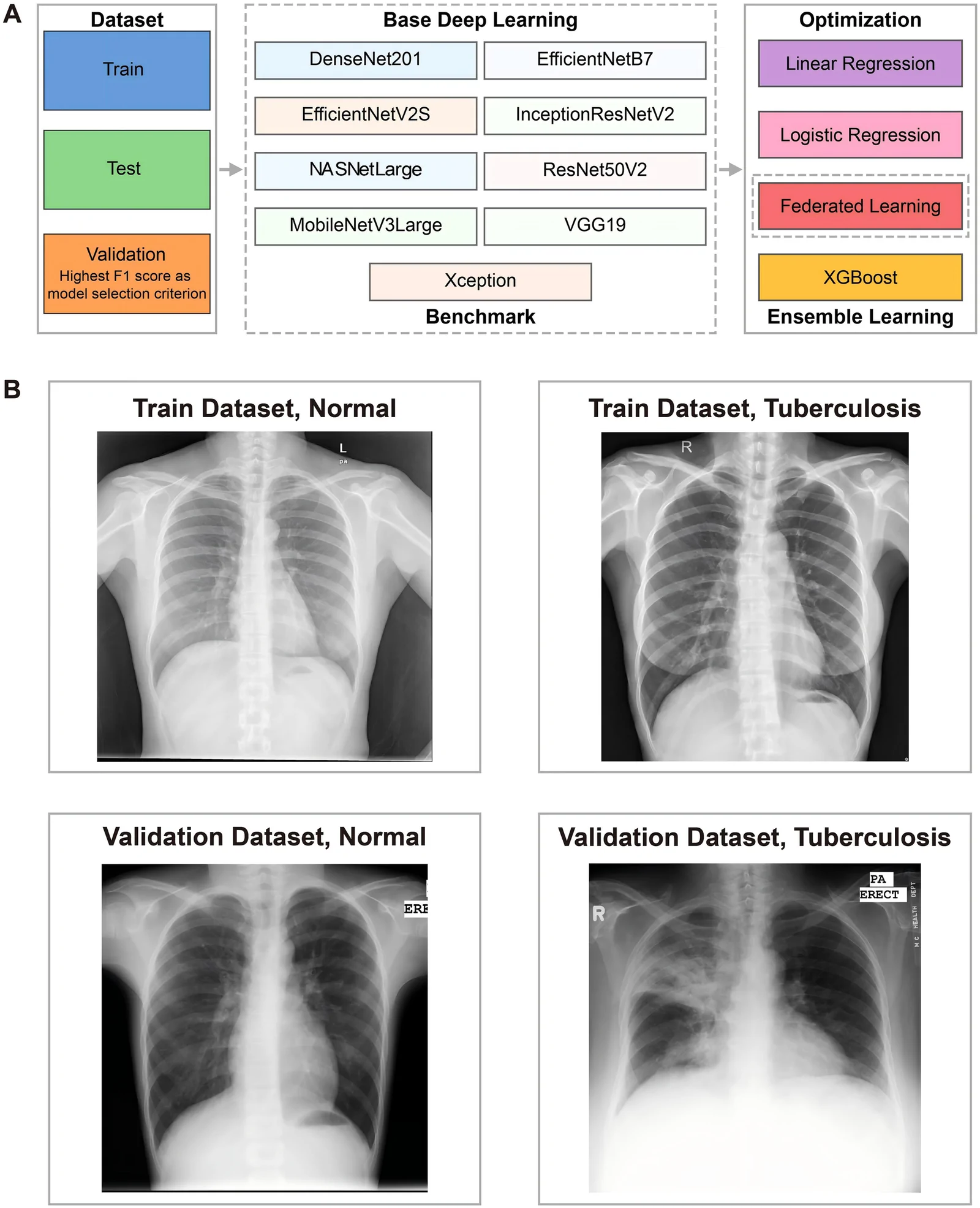
Research technical approach and representative cases of chest x-ray imaging of pulmonary tuberculosis. **(A)** Technical flow. **(B)** Representative cases of chest x-ray imaging of pulmonary tuberculosis.

The main workflow of this study is shown in Figure 1A. This study completed the construction and preprocessing of the CXR image dataset for PTB. The training dataset, internal test dataset and independent external validation dataset maintained clear case grouping. Representative images showed that CXR images of positive PTB cases could show typical signs, which differed from the clear lung structure of normal negative cases (Figure 1B).

**Figure 2:**
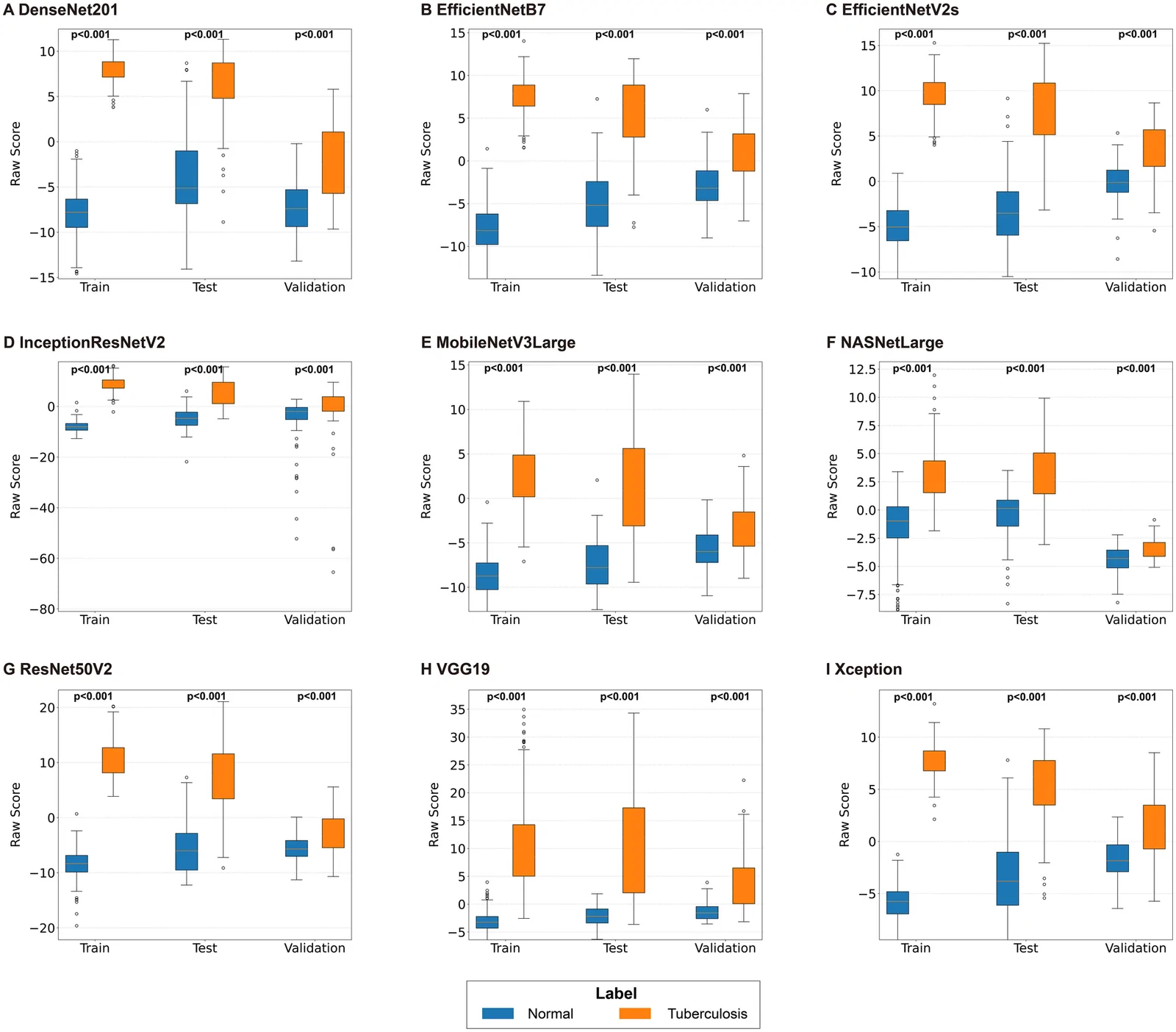
Inter-group differences in raw scores of basic deep learning models. **(A–I)** represent the original score distribution box plots for the DenseNet201, EfficientNetB7, EfficientNetV2S, InceptionResNetV2, MobileNetV3Large, NASNetLarge, ResNet50V2, VGG19, and Xception models, respectively. In the box plots, the boxes represent quartile intervals, the midline of the box represents the median, the whiskers represent the data range, and the scatter points represent outliers. Blue boxes represent normal samples, and orange boxes represent tuberculosis-positive samples. The *p*-values in the figures are the statistical results of the Mann–Whitney *U* test for inter-group differences, and the inter-group differences for all models are *p* < 0.001.

Based on the prediction results of nine single deep learning models, this study first verified the models' ability to distinguish between normal and PTB-positive samples. The Mann–Whitney *U* test results showed that the original prediction scores output by the nine convolutional neural network models all exhibited significant statistical differences between normal and PTB-positive samples (all *P* < 0.001; Figure 2). Specifically, the score distributions of normal and positive samples from models such as DenseNet201, EfficientNetB7, EfficientNetV2S, and Xception showed no significant overlap, demonstrating strong inter-group discrimination ability. Models such as InceptionResNetV2, MobileNetV3Large, and NASNetLarge showed a small amount of sample score overlap, but still managed to distinguish between the two groups of samples. These results confirm that the nine deep learning models selected in this study can effectively extract characteristic information from CXR images of PTB and possess good case discrimination ability, providing a foundation for subsequent benchmark performance testing and ensemble model construction.

**Figure 3:**
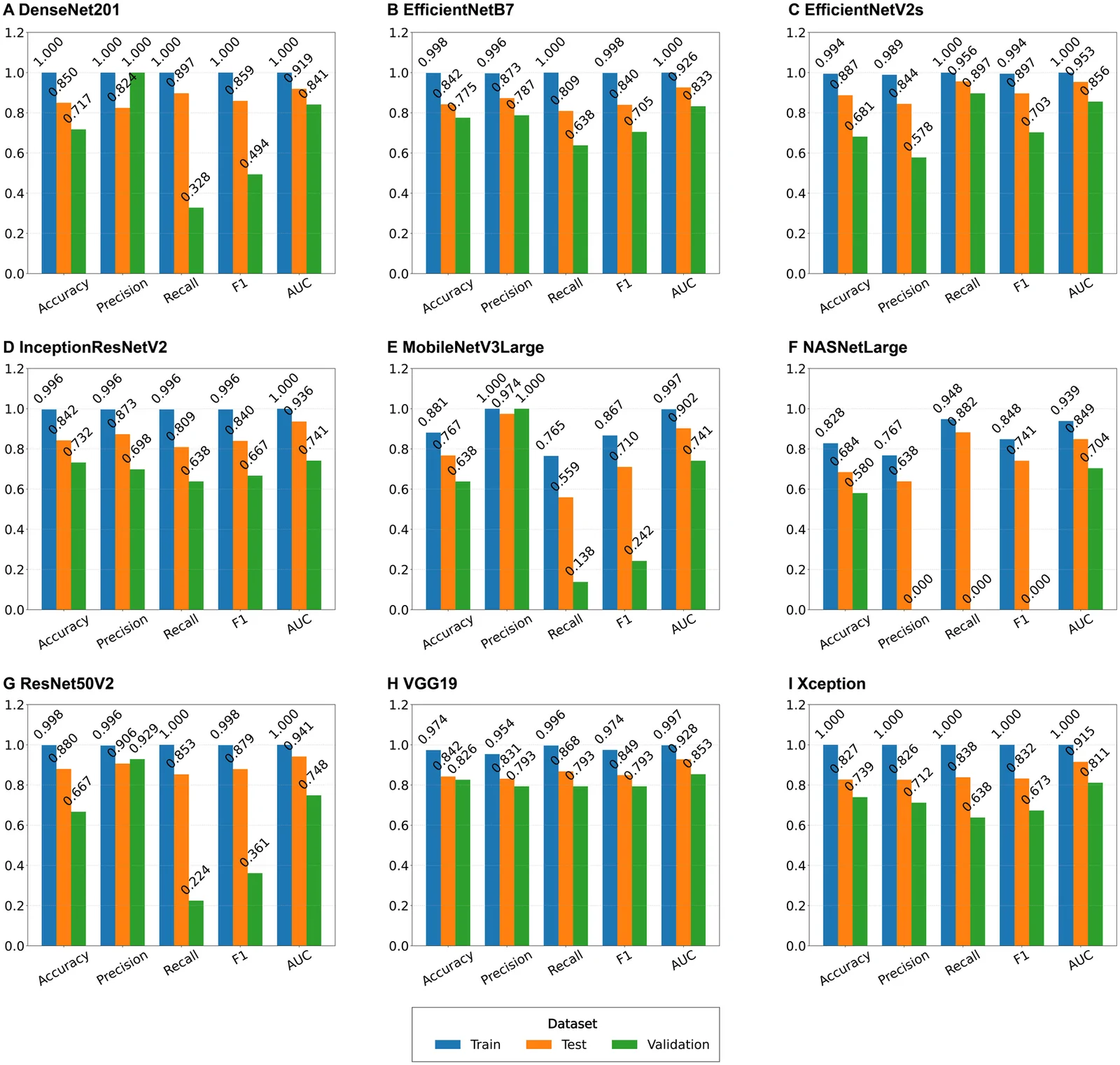
Classification performance of the basic deep learning model. **(A–I)** represent the classification metric bar charts for the DenseNet201, EfficientNetB7, EfficientNetV2S, InceptionResNetV2, MobileNetV3Large, NASNetLarge, ResNet50V2, VGG19, and Xception models, respectively.

This study benchmarked the performance of nine deep learning models, evaluating five classification metrics across training subsets, internal test subsets, and independent external validation datasets. Results show that all models exhibited excellent classification performance on the training subset, with accuracy, precision, recall, F1 score, and AUC all approaching 1.000, confirming their effective learning of PTB-related imaging features from the training data. On the internal test dataset, all models maintained good classification performance, with most models achieving an AUC greater than 0.800 and an F1 score greater than 0.850. The VGG19 model demonstrated the best validation performance, achieving an AUC of 0.853 and F1 score of 0.793 (Figure 3).

On the independent external validation dataset, all models showed varying degrees of performance degradation. Among them, the VGG19 model exhibited the best generalization performance, with a validation dataset accuracy of 0.826, precision of 0.793, recall of 0.793, F1 score of 0.793, and AUC of 0.853. It was the model among the nine single models to have both an F1 score and AUC nearly 0.800 on the validation dataset. The validation dataset AUCs of DenseNet201, EfficientNetB7, EfficientNetV2S, and Xception models were 0.741, 0.833, 0.856, and 0.811, respectively, and their F1 scores were 0.494, 0.705, 0.703, and 0.673, respectively, all demonstrating good generalization performance. However, the validation dataset performance of MobileNetV3Large and NASNetLarge models showed the most significant decline, with F1 scores of 0.242 and 0.000, and AUCs of 0.741 and 0.704, respectively, which may not meet the clinical needs for PTB identification.

**Figure 4:**
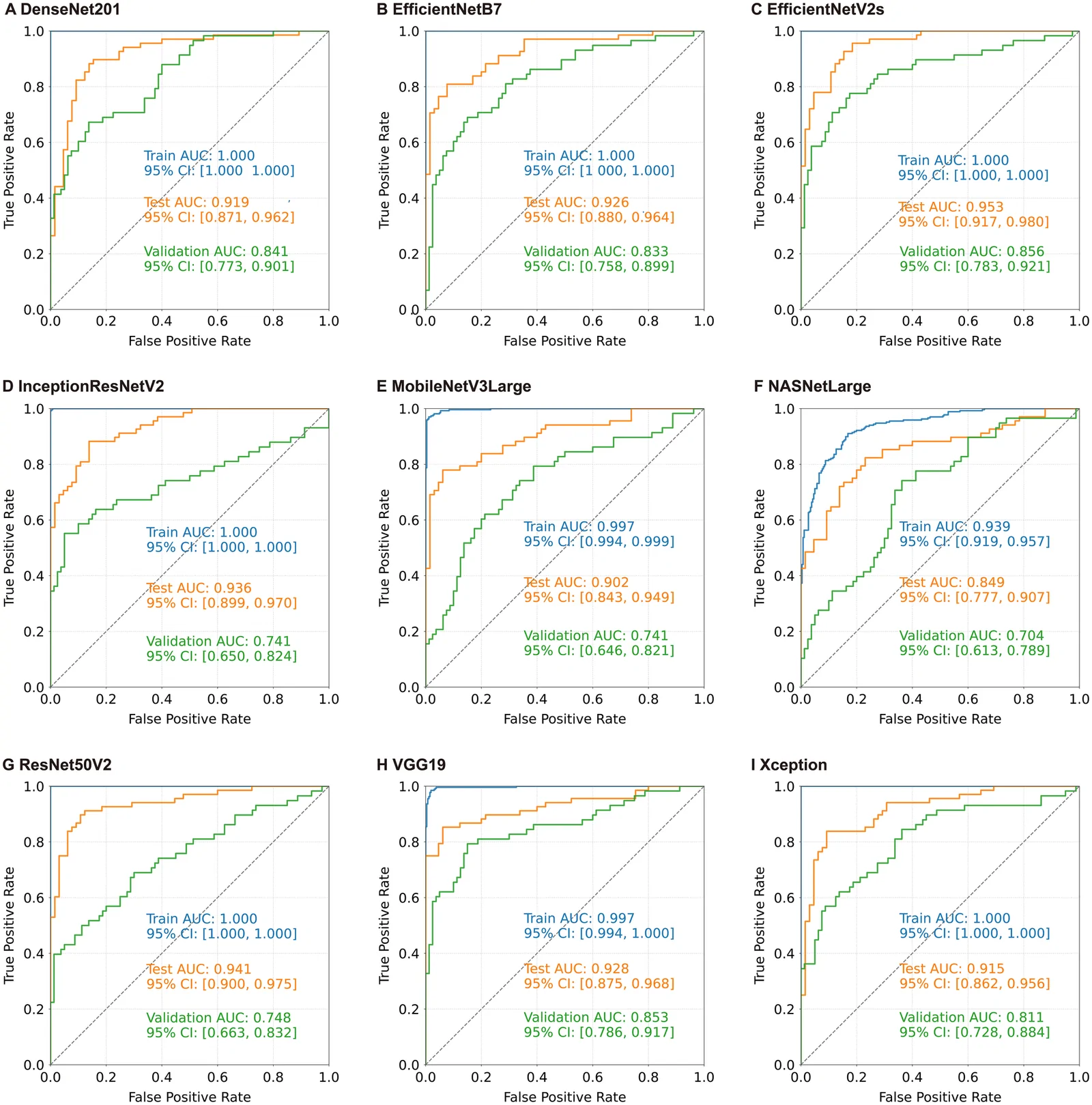
Receiver operating characteristic curves of the basic deep learning model. **(A–I)** represent ROC curves for the DenseNet201, EfficientNetB7, EfficientNetV2S, InceptionResNetV2, MobileNetV3Large, NASNetLarge, ResNet50V2, VGG19, and Xception models, respectively. The black dashed line represents the reference diagonal (AUC = 0.500). This figure marks the AUC values and 95% confidence intervals for each dataset. ROC, receiver operating characteristic; AUC, area under the curve.

ROC curve analysis further validated the classification performance of each model. The ROC curves of all models on the training dataset were close to the upper left corner, with AUC values close to 1.000% and 95% CIs not cross 0.5, confirming the training effect of the models. On the independent external validation dataset, the VGG19 model's ROC curve was closest to the upper left corner, with an AUC of 0.853 and a 95% CI of 0.786–0.917, demonstrating good generalization classification ability, consistent with the aforementioned metrics (Figure 4). These benchmark results clarified the advantages and disadvantages of different deep learning models in the CXR identification task of PTB, providing a basis for feature selection in subsequent ensemble models.

### 3.2 Performance optimization and evaluation of ensemble models

**Figure 5:**
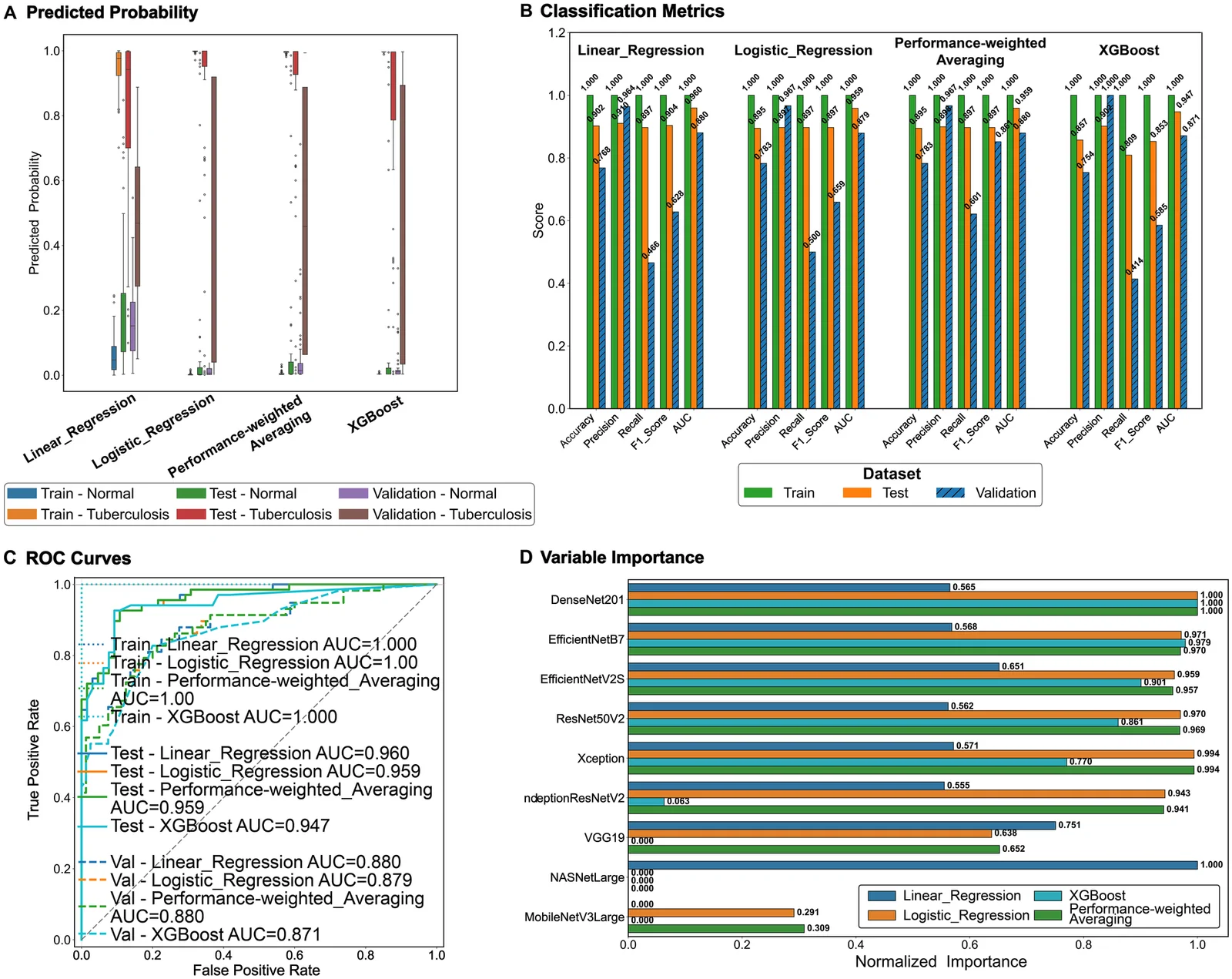
Classification performance of the ensemble model. **(A)** Predicted probability distribution of normal and tuberculosis samples by the ensemble model. **(B)** Comparison of classification performance metrics of the four ensemble models. **(C)** ROC curves of the four ensemble models; the black dashed line represents the reference diagonal (AUC = 0.500); the AUC values for each model's corresponding dataset are marked in the figure. **(D)** Importance of normalized variables for the nine base models. ROC, receiver operating characteristic; AUC, area under the curve; Val, validation.

Based on the original prediction scores of nine single models, this study constructed and evaluated the performance of four ensemble models. The results showed that the ensemble strategy can improve the classification performance and generalization ability of the PTB CXR identification model. Prediction probability distribution analysis showed that all four ensemble models could effectively separate normal and PTB positive samples. The prediction probability of positive samples was concentrated around 1.0, while the prediction probability of normal samples was concentrated around 0, with significant differences between groups. Among them, the performance-weighted averaging model had the best inter-group separation effect, with almost no overlap in sample probabilities (Figure 5A; Supplementary Table 1).

The comparison of classification metrics shows that all four ensemble models exhibit near-perfect classification performance on both the training and internal test dataset, with accuracy, precision, recall, F1 score, and AUC all reaching 0.900. On the independent external validation dataset, the performance-weighted averaging model demonstrates the best generalization performance, achieving an accuracy of 0.783, precision of 0.898, recall of 0.897, F1 score of 0.861, and AUC of 0.880. All metrics are superior to the best single model (VGG19) and the other three ensemble strategies. The logistic regression and XGBoost models show the second-best validation dataset performance. The linear regression model shows the smallest performance improvement (Figure 5B).

ROC curve analysis further confirmed the performance advantages of the ensemble model. The validation dataset ROC curves of all four ensemble models were superior to the best single model. The performance-weighted averaging model's ROC curve was closest to the top left corner, with an AUC of 0.880 and a 95% CI of 0.812–0.935, demonstrating the best classification specificity and sensitivity (Figure 5C). Variable importance analysis showed that single models with excellent generalization performance, such as DenseNet201, EfficientNetB7, EfficientNetV2S, and Xception, exhibited higher normalization importance in all four ensemble strategies, representing the core contributing factors to the improved performance of the ensemble models. Conversely, models with poor generalization performance, such as MobileNetV3Large and NASNetLarge, had variable importance close to 0 in some ensemble models, contributing no significant contribution to the final prediction results (Figure 5D). These results confirm that the performance-weighted averaging ensemble strategy can integrate the predictive advantages of each single model, avoid the performance shortcomings of single models, and improve the performance of CXR recognition for PTB, making it the optimal model selected in this study. Compared with existing results, our study evaluated nine basic deep learning models while using an ensemble strategy to achieve substantial PTB classification performance (Supplementary Table 2).

## 4 Discussion

TB is a major global public health problem, and insufficient screening capacity at the grassroots level is a core bottleneck restricting TB prevention and control. Chest x-rays, as a screening method for PTB, suffer from numerous problems such as low efficiency, poor consistency, and reliance on experience in manual diagnosis, necessitating intelligent auxiliary diagnostic tools. This study addresses this clinical need by constructing and validating a PTB CXR image recognition system based on a deep learning ensemble model. First, benchmark performance tests were conducted on nine mainstream convolutional neural network models to clarify the advantages and disadvantages of different models in PTB identification tasks. Then, the performance of four model ensemble strategies was constructed and compared, ultimately selecting the optimal ensemble model based on performance-weighted averaging. This model demonstrated excellent classification performance and generalization ability on independent external validation datasets, providing a viable intelligent tool for grassroots PTB screening.

This study first validated the performance of nine mainstream convolutional neural network models in CXR recognition of PTB. The results showed that all models could significantly distinguish between normal and positive PTB samples (all *P* < 0.001; Figure 2), confirming that deep learning models can effectively capture characteristic lesion information in CXR images of PTB, consistent with the conclusions of existing research (21). Several studies have already confirmed that CNN models can achieve a diagnostic level comparable to that (22, 23) of radiologists in CXR recognition of PTB, with models such as DenseNet, EfficientNet, ResNet, and Xception showing good application potential (24). The benchmark results of this study further expand on the conclusions of existing research (25). Under a unified training strategy and evaluation criteria, the performance of nine mainstream models was compared (26). It was found that the VGG19 model exhibited the best generalization performance on the independent external validation dataset, with both F1 score and AUC nearly reaching 0.8. In contrast, the MobileNetV3Large and NASNetLarge models showed poor generalization performance and could not meet the needs of clinical applications. This result differs somewhat from existing research, which suggests that lightweight MobileNet models perform well in TB identification (27). This discrepancy may be due to the fact that existing studies often train and validate models on single-center datasets without evaluating generalization performance using independent external datasets. Lightweight models (28), with fewer parameters and limited feature extraction capabilities, are more prone to performance degradation on heterogeneous data (29, 30). In TB CXR identification tasks, model selection should not only focus on performance on the training and internal test datasets but also emphasize the model's generalization ability on independent external data. Models such as Xception, DenseNet201, and EfficientNetV2S exhibit superior generalization performance and are more suitable for clinical translational applications in TB screening.

Model ensemble strategies can improve the classification performance and generalization ability of pulmonary TB CXR recognition models. Among these, the performance-weighted averaging demonstrated the best performance, achieving an F1 score and AUC above 0.8 on the independent external validation dataset (Figure 5B), outperforming the best single model and other ensemble strategies. This result aligns with existing research on model ensemble in medical imaging, which has confirmed that model ensemble can effectively integrate the feature extraction advantages of multiple base models, reduce the prediction variance of single models, and avoid the risk of overfitting, thereby improving the model's classification performance and robustness (31). This study further confirms this conclusion by comparing the performance of four ensemble strategies—linear regression, logistic regression, ensemble model, performance-weighted averaging, and XGBoost^22^ in the TB CXR recognition task. The weighted federated average algorithm was found to be the best performer. The underlying mechanism of this result lies in the fact that the federated learning strategy uses the generalization performance of each single model as the weight for weighted aggregation, giving higher weights to models with excellent generalization performance while reducing the weights of models with poor generalization performance. This avoids the performance shortcomings of some single models and maximizes the integration of the predictive advantages of each model (23, 32). In comparison, while logistic regression and XGBoost models can also achieve performance improvements, they are more prone to overfitting on small independent validation datasets, and linear regression models cannot capture the nonlinear relationships between features, resulting in limited performance improvements. The results of this study confirm the superior performance of the weighted average algorithm in CXR identification of PTB, providing a new methodological approach for model optimization in this field.

Independent external validation is crucial for evaluating the performance of deep learning models (33). In this study, all individual models exhibited near-perfect classification performance on both the training and internal test subsets, but all showed varying degrees of performance degradation on the independent external validation dataset. The MobileNetV3Large and NASNetLarge models showed the most significant performance degradation, even failing to meet basic classification requirements. This result suggests that validation based solely on a single-center dataset significantly overestimates the true performance of deep learning models, and models lacking independent external validation have extremely limited application value in real-world clinical scenarios. The core reason for this performance degradation is the difference in image acquisition equipment, scanning parameters, population characteristics, and lesion types across different datasets—a phenomenon known as “domain shift” in medical imaging. Models trained on a single center often learn irrelevant features relevant to the dataset, rather than the universal features related to PTB, leading to performance degradation on heterogeneous data (34, 35). Future research in this field should emphasize the application of independent external validation to ensure the rigor of model evaluation.

This study is innovative and clinically valuable. First, under unified standards, it compared the benchmark performance, particularly the generalization performance, of nine mainstream CNN models in CXR image recognition for PTB, providing a reference for model selection in this field. Second, it compared four different ensemble model, demonstrating the good performance of the weighted average algorithm in this task, offering new insights for optimizing AI diagnostic models for PTB. Third, it used independent external datasets for model validation, and the selected ensemble model exhibited high generalization ability, making it applicable to primary care PTB screening scenarios. Furthermore, the methodological framework of this study is universal and can be extended to CXR image recognition tasks for other chest diseases, providing a procedural reference for the construction of medical imaging AI models.

This study still has certain limitations. First, this study trained and validated the model based on a publicly available dataset. Although an independent external validation dataset was used, the sample size of the dataset is still relatively limited. Future research should incorporate multi-center, large-sample real-world data to further validate the model's performance and optimize its recognition capabilities across different populations and disease types. Second, this study only focused on the binary classification of PTB and did not further subdivide the identification of different lesion types, stages, and activity levels. Future research could further expand the model's functionality to achieve accurate classification and activity assessment of PTB, providing a reference for clinical treatment decisions.

Overall, the performance-weighted averaging ensemble model constructed in this study can identify CXR images of PTB, demonstrating generalization performance and clinical application potential. The findings provide a generalizable intelligent tool for primary-level PTB screening and also offer methodological references for the construction, optimization, and validation of medical imaging AI models. With future large-sample, multi-center clinical validation and optimization, this model may play a role in global PTB prevention and control efforts.

## 5 Conclusion

TB is a major infectious disease worldwide, and CXR examination is a primary screening method at the grassroots level; however, manual diagnosis may have limitations. This study aims to construct and validate a deep learning ensemble model-based PTB CXR image recognition system, providing an intelligent solution for PTB screening at the grassroots level. The Shenzhen PTB CXR dataset was used as the training dataset, divided into a training subset and an internal testing subset. The Montgomery dataset was used as an independent external validation dataset. Benchmark tests were performed on nine mainstream CNN models, and four ensemble models were further constructed to evaluate the classification performance and generalization ability of each model. The results show that all nine individual CNNs models can distinguish between normal and PTB samples, with the VGG19 model exhibiting the highest single-model generalization performance. The performance-weighted averaging ensemble model improves recognition performance, achieving an F1 score of 0.861 and AUC of 0.880 on the independent external validation dataset, outperforming the best single model and other ensemble strategies. The performance-weighted averaging model constructed in this study can identify PTB CXR images and possesses good generalization performance, providing an intelligent tool for PTB screening at the grassroots level. In the future, further prospective clinical validation with multiple centers and large samples can be carried out to promote the clinical translation and application of the model and help improve the efficiency of global PTB prevention and control.

## Data Availability

Publicly available datasets were analyzed in this study. This data can be found here: The datasets analyzed in this study are publicly available from the National Library of Medicine/NIH Tuberculosis Chest x-ray Datasets repository: https://data.lhncbc.nlm.nih.gov/public/Tuberculosis-Chest-x-ray-Datasets/. The datasets used were the Shenzhen Hospital CXR Set and the Montgomery County CXR Set. No accession numbers are available/applicable.
